# ATP1A1 is a promising new target for melanoma treatment and can be inhibited by its physiological ligand bufalin to restore targeted therapy efficacy

**DOI:** 10.1186/s12935-023-03196-y

**Published:** 2024-01-04

**Authors:** Laura Soumoy, Aline Genbauffe, Lena Mouchart, Alexandra Sperone, Anne Trelcat, Léa Mukeba-Harchies, Mathilde Wells, Bertrand Blankert, Ahmad Najem, Ghanem Ghanem, Sven Saussez, Fabrice Journe

**Affiliations:** 1 Laboratory of Human Anatomy and Experimental Oncology, Faculty of Medicine and Pharmacy, University of Mons (UMONS), 7000 Mons, Belgium; 2 Laboratory of Pharmaceutical Analysis, Faculty of Medicine and Pharmacy, University of Mons (UMONS), 7000 Mons, Belgium; 3 Laboratory of Clinical and Experimental Oncology, Institut Jules Bordet, Université Libre de Bruxelles (ULB), 1000 Brussels, Belgium; 4 Department of Otolaryngology and Head and Neck Surgery, CHU Saint-Pierre, 1000 Brussels, Belgium; 5 Institut National de la Santé et de la Recherche Médicale (INSERM) U981, Gustave Roussy Cancer Campus, Villejuif, France

**Keywords:** Melanoma, Resistance, ATP1A1, Sodium pump, Cardiotonic steroid, Bufalin

## Abstract

Despite advancements in treating metastatic melanoma, many patients exhibit resistance to targeted therapies. Our study focuses on ATP1A1, a sodium pump subunit associated with cancer development. We aimed to assess ATP1A1 prognostic value in melanoma patients and examine the impact of its ligand, bufalin, on melanoma cell lines in vitro and in vivo. High ATP1A1 expression (IHC) correlated with reduced overall survival in melanoma patients. Resistance to BRAF inhibitor was linked to elevated ATP1A1 levels in patient biopsies (IHC, qPCR) and cell lines (Western blot, qPCR). Additionally, high ATP1A1 mRNA expression positively correlated with differentiation/pigmentation markers based on data from The Cancer Genome Atlas (TCGA) databases and Verfaillie proliferative gene signature analysis. Bufalin specifically targeted ATP1A1 in caveolae, (proximity ligation assay) and influenced Src phosphorylation (Western blot), thereby disrupting multiple signaling pathways (phosphokinase array). In vitro, bufalin induced apoptosis in melanoma cell lines by acting on ATP1A1 (siRNA experiments) and, in vivo, significantly impeded melanoma growth using a nude mouse xenograft model with continuous bufalin delivery via an osmotic pump. In conclusion, our study demonstrates that ATP1A1 could serve as a prognostic marker for patient survival and a predictive marker for response to BRAF inhibitor therapy. By targeting ATP1A1, bufalin inhibited cell proliferation, induced apoptosis in vitro, and effectively suppressed tumor development in mice. Thus, our findings strongly support ATP1A1 as a promising therapeutic target, with bufalin as a potential agent to disrupt its tumor-promoting activity.

**Supplementary Information:**

The online version contains supplementary material available at 10.1186/s12935-023-03196-y.

## Introduction

Melanomas frequently harbor mutations in proteins of the MAPK pathway, leading to constitutive activation. The most commonly mutated proteins include BRAF (50%), NRAS (20–30%), NF1 (10–15%), and KIT (5–10%) [1]. Small molecule inhibitors targeting these mutant proteins have significantly improved the treatment of metastatic melanoma [2]. However, a major challenge is the development of resistance to these inhibitors, either innate or acquired within weeks or months of therapy initiation [3, 4]. Moreover, excepted the presence of the ^V600E^BRAF mutation, there are currently no validated markers to predict patient response to targeted therapy in melanoma [5, 6]. Therefore, two key challenges exist: identifying biomarkers for predicting response to current targeted therapies and discovering new targets to enhance response and delay or prevent resistance development.

Our research focuses on the sodium pump, which has recently been identified as a plasma membrane receptor involved in signal transduction, particularly in cancer [7]. The pump comprises three subunits (α, β, and γ), with the α-catalytic subunit, encoded by four isoforms (ATP1A1, ATP1A2, ATP1A3, and ATP1A4), primarily responsible for signal transduction [8]. The ATP1A1 isoform has been found to be overexpressed in various cancers [10–14], including melanoma [15, 16], compared to healthy tissues. Elevated ATP1A1 levels have been associated with advanced cancer staging, reduced recurrence-free survival (RFS), and overall survival (OS) in gastric cancer [17] and hepatocellular carcinoma [18]. Thus, we aimed to investigate ATP1A1 expression in melanoma samples to determine its association with patient survival and response to targeted therapy.

Otherwise, cardiotonic steroids such as digoxin, ouabain, and bufalin interact with ATP1A1 [19]. Epidemiological studies have shown that patients receiving cardiotonic steroids for heart disorders have a lower risk of breast cancer recurrence, renal tumor development, and leukemia [20]. Our study focuses on the cardiotonic steroid bufalin, as recent research has revealed endogenous bufalin expression in the serum of healthy individuals [21]. Additionally, serum bufalin levels were reduced in patients with hepatocellular carcinoma [21]. Bufalin has demonstrated various anti-cancer effects in different tumor types, including melanoma [22], but no study to date has combined bufalin with targeted therapy in metastatic melanoma [23].

## Materials and methods

### Patient samples

For the clinical study, a total of 84 skin and lymph node metastases were obtained from patients with stage III or IV melanoma who underwent surgery at Institut Jules Bordet (Brussels, Belgium) between 1998 and 2009. This study was conducted in accordance with the REMARK guidelines [24, 25] and approved by the ethics committee of Institut Jules Bordet (CE1959). The samples were registered in the Biobank of Institut Jules Bordet (BB190035). The clinical characteristics of the patients are summarized in Table 1. It is worth noting that the median overall survival (OS) of the patients was 4.85 years, ranging from 0.8 to 28.4 years.

**Table 1:** Characteristics of melanoma patients and tumors used to evaluate ATP1A1 protein expression by IHC (n = 84)

Parameters	N	Missing values	Median	Range
Gender (female/male)	50/34	0		
Breslow thickness (≤1/>1)	19/65	0		
Ulceration of primary (yes/no)	29/55	0		
Lymph node invasion (1/≥1)	40/23	21		
Overall survival (years)	80	4	4.85	0.8–28.4
Status (alive/dead)	26/58	0		

Additionally, biopsies were collected from eight patients with metastatic melanoma carrying ^V600E^BRAF mutations. These biopsies were snap-frozen in liquid nitrogen prior to the initiation of vemurafenib therapy. Among the patients, four demonstrated a response to treatment, with either a partial or complete response as the best response according to the Response Evaluation Criteria In Solid Tumors (RECIST) 1.1 [26] or the PET Response Criteria in Solid Tumors (PERCIST) [27]. The four other patients did not respond to the treatment and exhibited progressive disease as the best response.

### Immunohistochemistry

**Fig. 1:**
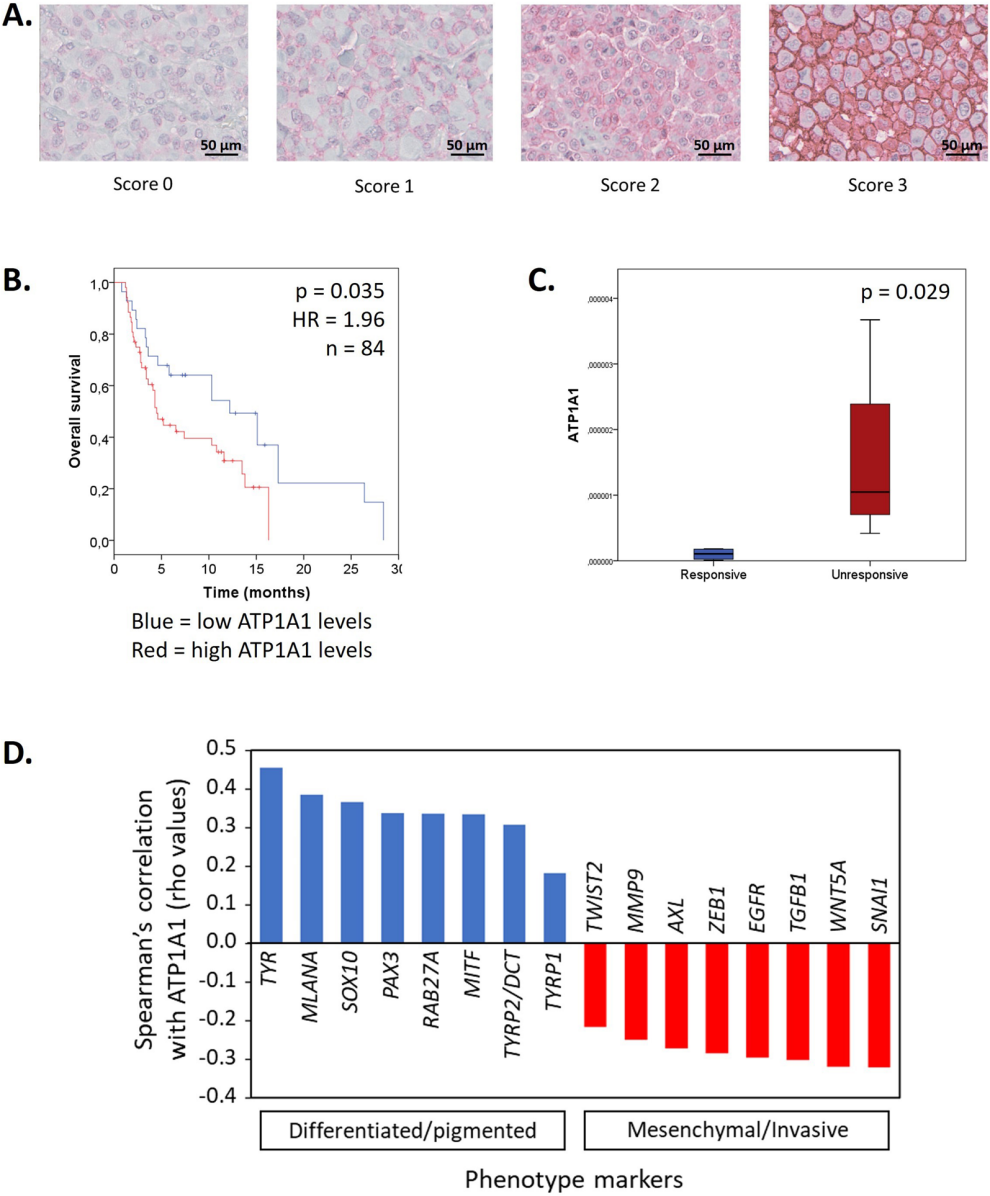
ATP1A1 expression in samples from melanoma patients. **A** Representative IHC of ATP1A1 in melanoma metastases. Examples of staining intensity scored at 0 (−), 1 (+), 2 (+ +) and 3 (+ + +) (scale bars = 50 µm). **B** Kaplan–Meier curves comparing overall survival between groups with low (blue) and high (red) ATP1A1 protein levels (Cox regression). **C** ATP1A1 mRNA levels in 8 patient biopsies collected prior targeted therapies and defined as responders (blue) or non-responders (red) to the BRAF inhibitor vemurafenib (Mann–Whitney test). **D** Evaluation of the correlation between ATP1A1 and a panel of phenotype markers of pigmented versus invasive melanomas, 448 cutaneous melanomas were investigated using the cBioPortal for Cancer Genomics (http://www.cbioportal.org/) based on TCGA, PanCancer Atlas data sets. All correlations are significant (p < 0.001, Spearman rho test)

IHC using a mouse monoclonal antibody raised against ATP1A1 (M7-BP-E9, ThermoFisher Scientific) was performed on a BenchMark XT System (Ventana, Tucson, AZ, USA). The detection of primary antibody was executed using the ultraView Universal Alkaline Phosphatase Red Detection Kit (Ventana). Immunostaining was evaluated with an Axio-Cam MRC5 optical microscope (Zeiss, Hallbergmoos, Germany) and a score from 0 to 300 was calculated by adding the percentage of tumour cells with none (= 0), weak (= 1), intermediated (= 2) or strong (= 3) staining intensity (Fig. 1A).

### Effectors

The BRAF inhibitor Dabrafenib, used for BRAF mutated cells, and the MEK inhibitor Pimasertib, used for NRAS mutated cells, were from Selleck Chemicals (Houston, TX, USA). The tyrosine kinase inhibitor Dasatinib, used for cKIT mutated cells, was from Bristol-Myers (New York, NY, USA). Bufalin was from PhytoLab (Vestenbergsgreuth, Germany). Methyl-β-cyclodextrin is from Merck (Burlington, MA, USA).

### Cell culture

The cell lines were derived from melanoma metastasis by the Laboratory of Clinical and Experimental Oncology at the Jules Bordet Institute (Brussels, Belgium). We worked with three cell lines harbouring different mutations and sensitive to specific targeted therapy (MM074 (V600EBRAF), MM161 (Q61RNRAS) and HBL (D820YcKIT)) and their corresponding cells with acquired resistance to the drugs (-R) developed by chronic treatment with increasing concentrations of the inhibitors. Cells were grown in Ham-F10 medium (Lonza, Bâle, Switzerland) supplemented with 10% fetal bovine serum and 1% penicillin/streptomycin (both from Life Technologie, Carlsbad, CA, USA). Cells were confirmed to be free of mycoplasma contamination by qPCR analysis.

### qPCR

mRNAs were extracted from slices of patient frozen tissues or cell pellets using the InnuPrep RNA mini kit 2.0 (Westburg Life Sciences, Westburg, Germany) and dosed using the Biodrop µlite (ThermoFisher Scientific). The retrotranscription was performed using the Maxima First Strand cDNA Synthesis Kit (ThermoFisher Scientific). The qPCR was completed using the SYBR green (Takyon Rox SYBR Core Kit Blue dTTP, Eurogentec, Liège, Belgium). The primers 18S (Fw: CATTTAGGTGACACTATAGAAGACGATCAGATACCGTCGTAGTTCC; Rv: GGATCCTAATACGACTCACTATAGGCCTTTAAGTTTCAGCTTTGCAACC), ATP1A1 (Fw: GGCCTTTAAGGTTGGACGTG; Rv: CACAGTAACATTGAGAACCCCC), ATP1A2 (Fw: TCTATCCACGAGCGAGAAGAC; Rv: CCATGTAGGCATTTTGAAAGGC), ATP1A3 (Fw: AAGGAGGTGGCTATGACAGAG; Rv: GTGAGTGCGTTAGGCCCAT) and ATP1A4 (Fw: AAGTTGTGGAGTTCACATGCC; Rv: GCTGG AAAAGTGAGTTGCGG) were from IDT-DNA (Iowa, USA). Ten µl were dispensed into each well of a 96-well plate (Microplate for PCR, 96 wells, with sealing film, 732–1591 VWR). Then, 2 µl of cDNA were added to each well. The plate was finally introduced into the thermocycler (Light-Cycler 96 FW13083, Roche). The program was launched according to the following cycle: preincubation 10 min at 95 °C, 2 steps amplification 15 s at 95 °C and 1 min at Tm (62 °C) repeated 40 times, and then the melting curve 15 s at 95 °C, 1 min to Tm (62 °C), 95 °C continuous acquisition. Raw data are analysed with LightCycler^®^ 96 SW 1.1 software. Data with a Cq error greater than 1 are discarded. Fold change (2^−ΔCT^) are calculated using the housekeeping gene 18S as a reference.

### Western blot

Proteins were extracted by cell lysis using Mammalian Protein Extraction Reagent and Protease & Phosphatase Inhibitor Cocktail 100x (both from Pierce, Rockford, IL, USA) and dosed using the BCA Pierce Protein Assay (ThermoFisher Scientific, Rockford, IL, USA). Then, 20 µg of proteins were charged on a 4–20% polyacrylamide gel (Bio-Rad Laboratories, Hercules, CA, USA) and migrate at 120 Volt for 1 h. The transfer on the nitrocellulose membrane was performed using the iBlot 2 Dry Blotting system (ThermoFisher Scientific). The primary anti-ATP1A1 (mouse, dilution 1:1000) (ThermoFisher Scientific), anti-phospho-Src (Tyr416 and Tyr527) (rabbit, dilution 1:1000) (Cell Signaling Technology, Danvers, MA, USA) and anti-β-actin (mouse, dilution 1:10000) (Novus Biologicals, UK) and secondary anti-mouse (Merck) antibodies were diluted in TBS-Tween 0.1% BSA 5%. Detection was performed using the NovexTM ECL Chemiluminescent Substrate Reagent Kit (Invitrogen, Carlsbad, CA, USA). The revelation was performed using either photographic films in a dark room and quantified using ImageJ or a LAS-3000 CCD camera (Fujifilm, Tokyo, Japan). Immunoreactive spot intensities were quantified using the software AIDA® Image Analyser 3.45 (Raytest®, Straubenhardt, Germany).

### Phosphokinase array

A Human Phospho-Kinase Array kit (R&D Systems, Minneapolis, MN, USA) was used to evaluate the variations of phosphorylation sites in 37 kinases in accordance to the manufacturer’s recommendations. The revelation was performed using a LAS-3000 CCD camera (Fujifilm). Immunoreactive spot intensities were quantified using the software AIDA® Image Analyser 3.45 (Raytest^®^).

### Immunofluorescence

Cells were fixed in paraformaldehyde 4% (Sigma Aldrich, Saint-Louis, MO, USA) and blocked in PBS 1x (Klinipath, Olen, Belgium) containing 0.3% TritonTM X-100 and 5% normal goat serum (Cell Signaling Technology). The cells were incubated overnight at 4 °C with antibody anti-ATP1A1 (M7-BP-E9, mouse, dilution 1:400) (ThermoFisher Scientific) and anti-Cav1 (3238, rabbit, dilution 1:200) (Cell Signaling Technology) diluted in PBS 1x, 0.3% TritonTM X-100 and 1% BSA (Sigma). The secondary antibodies were, respectively, Alexa FluorTM 555 Goat AntiMouse IgG and Alexa FluorTM 488 Goat Anti-Rabbit IgG (Invitrogen). Nucleus were stained using VectaShield-DAPI (Vector laboratories). Images were acquired using a confocal microscope (Nikon Ti2 A1RHD25, Tokyo, Japan).

### Proximity-ligation assay

Cells were fixed in paraformaldehyde 4% (Sigma) and blocked in Duolink Blocking solution 1x (Sigma Aldrich, Saint-Louis, MO, USA) for 1 h at 37 °C in a humidified chamber. The primary antibodies (see Immunofluorescence) were incubated overnight at 4 °C. Cells were then incubated with the PLA probes (Duolink In Situ PLA, probe and anti-rabbit PLUS and anti-mouse MINUS, Sigma Aldrich, Saint-Louis, MO, USA) for 1 h at 37 °C. Then ligation was performed for 40 min at 37 °C using the Duolink ligase and ligase buffer (Sigma). Amplification was performed using polymerase and Duolink Amplification Green and Orange 5x (Sigma). For the rest of the protocol, see Immunofluorescence.

### Clonogenic assay

To evaluate colony formation, 2000 cells per well were plated in 6-well plates. Cells were treated with bufalin for 2 weeks 24 h after plating. The media was refreshed once during the 14 days of growth culture. Then the cells were fixed with paraformaldehyde 4% and stained with 4% crystal violet solution (Sigma) for 30 min. The excess of crystal violet was removed with tap water and the colonies were counted.

### Cell proliferation

Cell proliferation assays were performed in 96-well plates (Sarstedt, Belgium). Cells were plated at 1500 cells/wells in 100 µl of culture medium. The next day, cells were supplemented with 100 µl of fresh medium containing bufalin or with vehicle for control. After 72 h, cells were washed and then fixed with 1% glutaraldehyde (Sigma) during 15 min. After washing, cells were stained with 4% crystal violet (Sigma) during 30 min, washed with tap water and leave on a bench at open air to dry. Cells were finally permeabilized with a solution of TritonTM X-100 (Sigma) during 90 min and the absorbance was determined at 570 nm with a spectrophotometer (VERSA max-SoftMax Pro, Molecular Devices, USA). Results were normalized to control (untreated cells).

### Apoptosis analysis

Cell apoptosis was performed by flow cytometry using the Muse^®^ Cell Analyzer (Merck) and the Muse Annexin V & Dead Cell Assay Kit (Luminex, Austin, TX, USA). Cell suspension is analysed using the MuseTM Cell Analyzer (Millipore Corporation, Burlington, USA).

### RNA interference

siRNAs silencing ATP1A1 (s1719, ThermoFisher Scientific) were transfected into cells using Lipofectamine 2000 Transfection Reagent (Invitrogen). The knock-down efficiency was assessed by qPCR and western blot 48 h after transfection as described above.

### Animal study

For this research, we used 24 nude mice (Male CR ATH HO MOUSE 28–34 days, Charles River, Saint-Germain-Nuelles, France). 5 × 10^–6^ MM074 or MM074-R cells diluted in 50/50 saline-Cultrex Basement Membrane Extract, Type 3 (R&D Systems) were injected subcutaneously into the right flank of the mice. One week after injection, mice were anesthetized with isoflurane and implanted with an osmotic pump (Alzet pump 1002, Alza corporation, Cupertino, CA, USA) containing bufalin diluted in saline DMSO 5%. The pump allows the stable administration of 2 mg/kg of bufalin each day for 14 days. The mice weight, general behaviour and the tumour size were monitored every 2 days. Tumour volume was calculated according to the formula (length x width x thickness)/2. After 2 weeks of treatment, the animals were sacrificed by lethal IP injection of Dolethal 200 mg/mL (pentobarbital sodium, Vetoquinol, Aartselaar, Belgium). Tumours, hearts, lungs, livers, kidneys and spleens were collected, fixed in Bouin and paraffined for HE staining and histological analyses. All animal experiments were performed according to the institutional guidelines and approved by the ethics committee of the University of Mons (Mons, Belgium) (SA-06-01).

### Statistical analysis

Statistical analyses were performed using IBM SPSS Statistics 21 software (IBM, Ehningen, Germany). At least 3 independent samples were compared using Mann–Whitney or ANOVA and Tukey post-hoc test, depending on normality of the distribution. The correlation between two continuous variables was assessed using the non-parametric Spearman rho test. A p-value < 0.05 was considered as statistically significant (* = p ≤ 0.05; ** = p ≤ 0.01; *** = p ≤ 0.001). For all experiments, a minimum of 3 replicates were performed. Animal data were presented in box plot (median and percentiles 5/25/75/95) and analyzed by Mann Whitney test. For the study of the patient survival curve, OS analyses were performed using Kaplan–Meier test. Cox regression models were applied to calculate the hazard ration (HR), 95% confidence interval and significance.

## Results

### ATP1A1 is overexpressed in melanoma and is associated with a poor survival

Firstly, we analyzed a TCGA (The Cancer Genome Atlas) dataset using The Human Protein Atlas [28] and identified that ATP1A1 mRNA is significantly overexpressed in melanoma compared to 16 other cancer types (Additional file 1: Fig. S1). Subsequently, we assessed the protein expression of ATP1A1 using immunohistochemistry (IHC) in 84 skin and lymph node metastases (Table 1) and observed varying intensities of ATP1A1 staining on the cell membranes, which were ranked on a scale of 0 to 3 (Fig. 1A). We evaluated the percentage of cancer tissue with staining intensity categorized as follows: no = 0, low = 1, medium = 2, high = 3. This established a scale ranging from 0 to 300, which we then divided by 3 to obtain a percentage score ranging from 0 to 100%. We searched for the optimal cut-off value by testing various percentiles from 20 to 80 by step of 5 and, finally dividing the population at 55% in low vs high ATP1A1 groups. We demonstrated that high ATP1A1 protein expression was significantly associated to shorter patient OS and, consequently, to a poor prognosis (Fig. 1B).

### ATP1A1 is predictive of resistance to BRAF inhibitor in patient samples

Furthermore, we investigated the expression of the ATP1A1 gene in 8 metastatic melanoma patients harbouring the ^V600E^BRAF mutation, which is the most common mutation in melanoma. Importantly, these samples were obtained prior to treatment with the BRAF inhibitor vemurafenib. We observed a significantly higher expression of ATP1A1 mRNA in patients who did not respond to vemurafenib compared to those who showed a response (Fig. 1C). Specifically, the expression of ATP1A1 was approximately 18-fold higher in tumor samples from non-responders compared to responders. Additionally, we examined the gene expression of other isoforms of the Na + /K + -ATPase α pump in these patients; however, we did not observe significant differences between responders and non-responders (Additional file 2: Fig. S2). These findings suggest that elevated ATP1A1 levels may serve as a specific predictive marker for resistance to BRAF inhibitor therapy.

### High ATP1A1 level correlates with a differentiated phenotype both in patients and cell lines

To further characterize the role of ATP1A1 in melanoma, we conducted an analysis using the cBioPortal for Cancer Genomics (http://www.cbioportal.org/) based on data from The Cancer Genome Atlas (TCGA) of 448 cutaneous melanoma samples from the PanCancer Atlas. Our analysis revealed a positive correlation between high ATP1A1 mRNA expression and a panel of markers associated with differentiation and pigmentation, including MITF, TYR, TYRP1, TYRP2/DCT, RAB27A, SOX10, PAX3, and MLANA (Fig. 1D) [29]. On the contrary, we observed a negative correlation between ATP1A1 expression and markers associated with a mesenchymal/invasive phenotype, such as AXL, EGFR, ZEB1, WNT5A, TGFβ1, SNAI1, MMP9, and TWIST2. These findings provide further evidence of the involvement of ATP1A1 in melanoma and its association with specific molecular markers related to cellular differentiation and pigmentation.

**Fig. 2:**
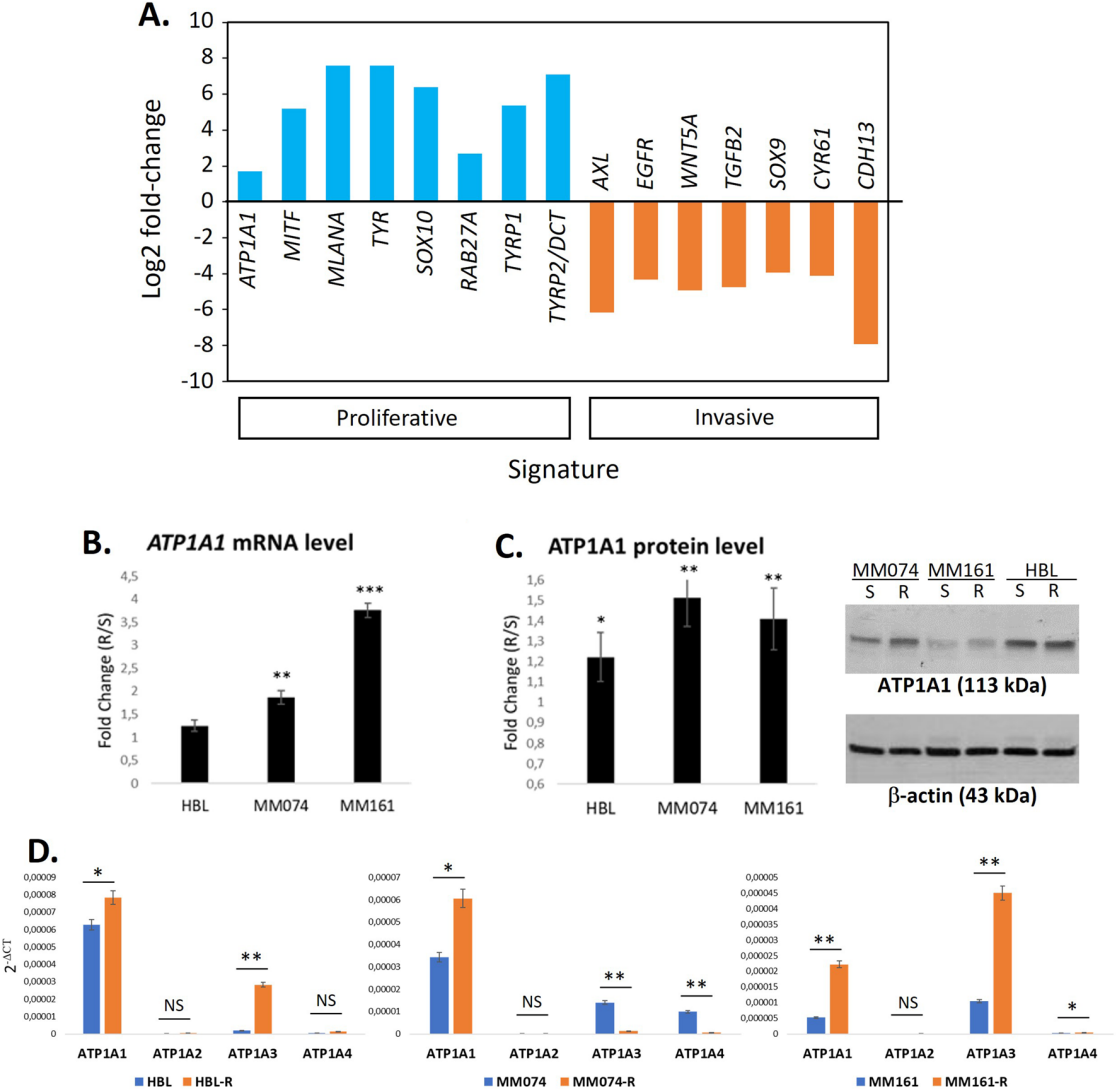
ATP1A1-4 expression in melanoma cell lines. **A** Evaluation of transcriptome profiling in 11 melanoma cell lines, focusing on ATP1A1 in relation with the Verfaillie proliferative/Invasive gene signatures. **B** Fold change between the ATP1A1 mRNA level in resistant cells and in their sensitive counterparts obtained by qPCR. **C** Fold change between the ATP1A1 protein level in resistant cells and in their sensitive counterparts obtained by Western blot. **D** ATP1A1 was the most expressed isoform in both sensitive and resistant MM074 and HBL cells, comparing the 4 isoforms (1–4); MM161 and MM161-R lines expressed lower levels of ATP1A1 and had also a higher level of ATP1A3. Data B-D are presented in mean + SD, ANOVA and Tukey post-hoc test

Additionally, examining the RNA-seq database and transcriptome profiling of 11 melanoma cell lines established by the Bordet team [30], we discovered that ATP1A1 is part of the Verfaillie proliferative gene signature, which is associated with the melanocytic state. This gene signature includes key transcription factors involved in melanocyte lineage, such as MITF and SOX10, as well as downstream markers like TYR, TYRP1, and MLANA, all of which play a role in cell differentiation and pigmentation. In contrast, ATP1A1 displays a strong negative correlation with the Verfaillie invasive gene signature (Fig. 2A) (p < 0.005). These findings collectively provide further evidence that ATP1A1 is predominantly expressed in differentiated and pigmented melanoma cell lines, consistent with the observations made in melanoma patients mentioned above.

### High ATP1A1 expression is associated with acquired resistance to targeted therapy in cell lines

To further validate the involvement of ATP1A1 in resistance to BRAF-targeted therapy, we conducted experiments using three different models of melanoma cell lines (harbouring mutations in cKIT, BRAF or NRAS). Our findings consistently demonstrated a significant increase in ATP1A1 expression in cell lines that had acquired resistance to targeted therapies, respectively dasatinib, vemurafenib, or pimasertib, compared to their sensitive counterparts. This increase was observed at both the mRNA and protein levels (Fig. 2B, C). Furthermore, among the isoforms of the Na + /K + -ATPase α pump, ATP1A1 was found to be the most highly expressed in both the sensitive and resistant MM074 and HBL cell lines, comparing the four isoforms (ATP1A1-4) (Fig. 2D). It is worth noting that the MM161 and MM161-R cell lines also exhibited high levels of ATP1A3 expression. This could be due to either upregulation of the gene or stabilization of the protein. However, there is no relationship between ATP1A3 and the sensitivity of bufalin or the resistance status to MAPKi of the cells. This supports that ATP1A1 is more likely to drive the apoptotic effect of bufalin. These results provide further evidence of the association between elevated ATP1A1 expression and resistance to targeted therapies, and highlight the predominant role of ATP1A1 among the isoforms in the tested cell lines.

### ATP1A1 is colocalized with caveolin-1 in melanoma cell lines

**Fig. 3:**
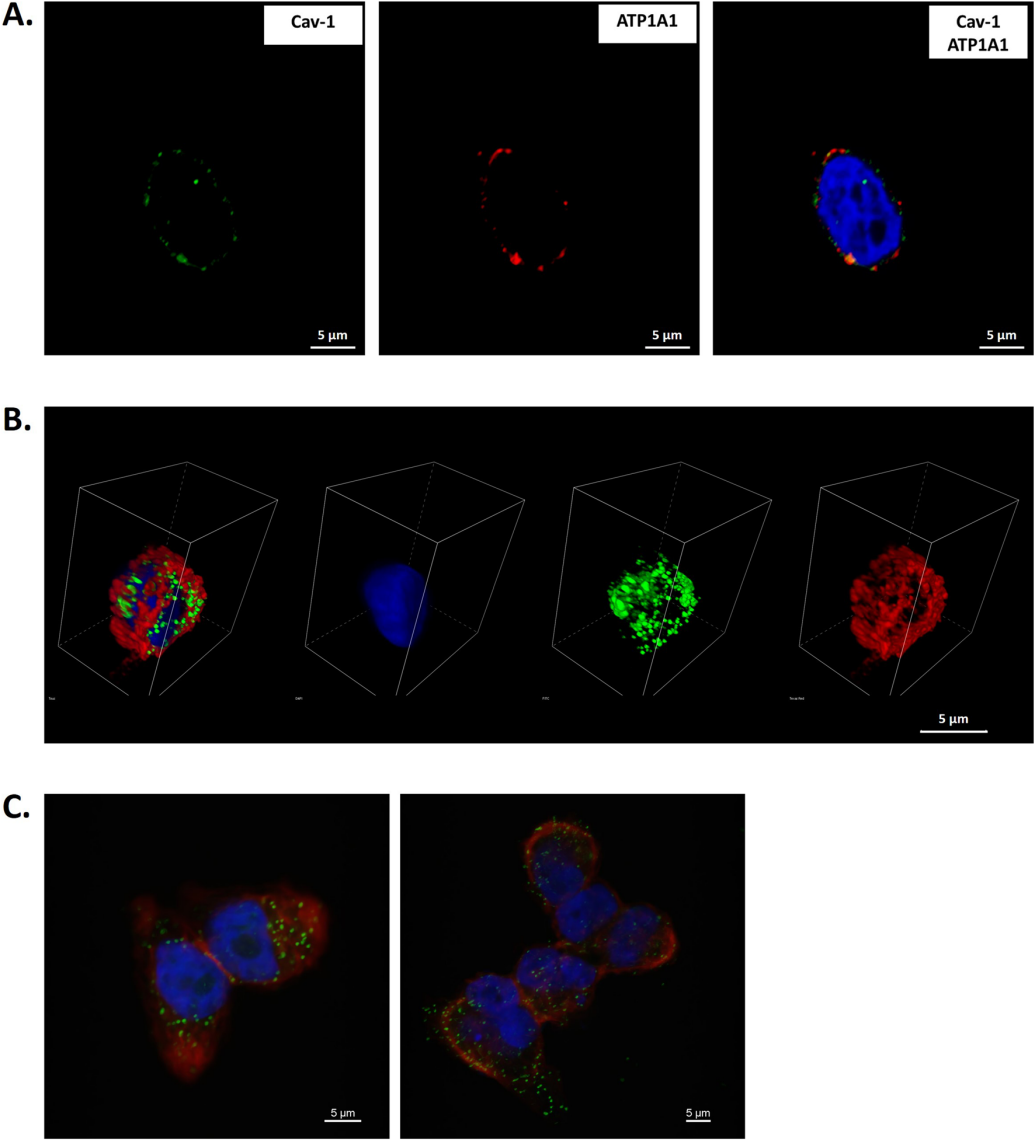
Confocal microscopy of ATP1A1 in melanoma cells. **A**, **B**. Immunofluorescence images of ATP1A1 (red) and Cav1 (green) in HBL cells (nucleus in blue); **a** colocalisation of ATP1A1 and Cav-1 is observed in 2D (**A**) and 3D (**B**) images. **C** The colocalization was confirmed by PLA; the green spots indicate a physical interaction between the two proteins, nuclei are stained in blue with DAPI whereas phalloidin was used to stain cell cytoplasm in red. Scale bars = 5 µm)

To assess the subcellular localization of ATP1A1 in melanoma cells, we conducted immunofluorescence and confocal microscopy analyses. Our observations indicated that ATP1A1 predominantly localizes to the caveolae of cell membranes. Additionally, through dual immunofluorescence staining, we observed a significant colocalization of ATP1A1 with Cav-1, a marker protein for caveolae (Fig. 3A, B). The physical interaction between ATP1A1 and Cav-1 was further confirmed using a proximity ligation assay, which demonstrated their close proximity and potential association (Fig. 3C). These findings provide strong evidence for the specific localization of ATP1A1 in caveolae and its interaction with Cav-1 in melanoma cells.

### Bufalin induced apoptosis of melanoma cells by acting on ATP1A1 in caveolae

**Fig. 4:**
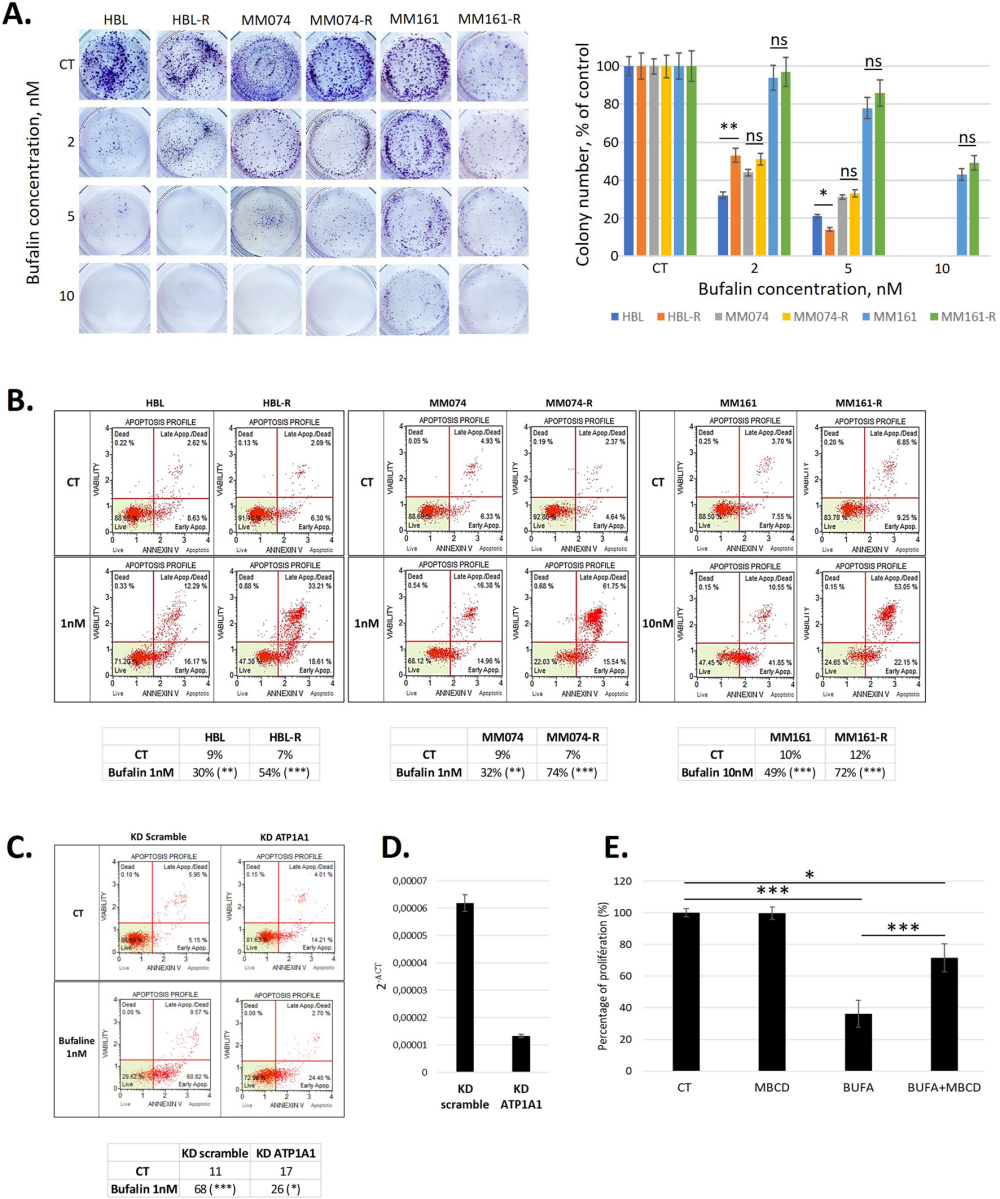
Bufalin inhibits colony formation and induces apoptosis through ATP1A1. **A** Clonogenic assays evaluating the effects of bufalin at 2, 5 or 10 nM. **B** Effect of bufalin (1 or 10 nM) on apoptosis induction. **C** Effect of silencing of ATP1A1 expression using siRNA in MM074-R line on the induction of apoptosis by 1 nM bufalin. **D** Confirmation of the knock-down of ATP1A1 by qPCR. **E**. Effect of caveolae disruption using methyl-beta-cyclodextrin (MBCD) in MM074-R line on the inhibition of cell proliferation by 1 nM bufalin and evaluation by crystal violet staining. All data are presented in mean + SD, ANOVA and Tukey post-hoc test

Given the significant role of ATP1A1 in melanoma progression and resistance to targeted therapy, we investigated the impact of bufalin, a ligand of ATP1A1, on cell proliferation using crystal violet assays. Interestingly, the resistant melanoma cell lines exhibited higher sensitivity to the inhibitory effects of bufalin compared to their respective sensitive counterparts (Table 2). Furthermore, we identified a significant negative correlation between the IC50 values for bufalin and the mRNA levels of ATP1A1 across the six cell lines (rho = -0.943, p = 0.005, Spearman correlation). To assess the effect of bufalin on colony formation, we conducted clonogenic assays and observed a dose-dependent reduction in colony numbers upon treatment with nanomolar concentrations of bufalin (Fig. 4A). Moreover, we conducted flow cytometry analyses using Annexin V to evaluate the induction of apoptosis by bufalin. Remarkably, bufalin was found to induce apoptosis in both sensitive and resistant cell lines. Notably, the resistant cell lines, which expressed higher levels of ATP1A1, exhibited a more pronounced increase in apoptosis rate (Fig. 4B). These results highlight the potential of bufalin to inhibit cell proliferation and induce apoptosis in melanoma cell lines, with a greater impact observed in the resistant lines that displayed elevated ATP1A1 expression.

**Table 2:** Expression of ATP1A1 mRNA (2^−CT^) in 6 cell lines (sensitive and resistant (-R) to MAPKi) and IC50 for bufalin in these cell lines

Lines	ATP1A1 mRNA	Ic50 bufalin (nM)
HBL	0.000063	2.10
HBL-R	0.000078	1.20
MM074	0.000034	3.40
MM074-R	0.000031	2.40
MM161	0.000005	9.10
MM161-R	0.000022	9.60

To confirm the dependency of these effects on ATP1A1, we used siRNA to silence ATP1A1 expression in the BRAF-mutated MM074-R cell line. The knock-down efficiency was verified by qPCR, which demonstrated a significant reduction in ATP1A1 mRNA levels (Fig. 4D). Subsequently, we evaluated the induction of apoptosis following bufalin treatment in cells transfected with ATP1A1 siRNA compared to cells transfected with scramble siRNA. The results revealed a notable decrease in apoptosis induction in ATP1A1 siRNA-transfected cells compared to the scramble siRNA-transfected cells (Fig. 4C). This clearly indicates that the pro-apoptotic activity of bufalin is mediated through its targeting of ATP1A1.

Furthermore, to demonstrate that bufalin acts as an anti-cancer agent specifically when ATP1A1 is located in caveolae, we used methyl-β-cyclodextrin (MBCD) to disrupt caveolae formation. Remarkably, when cells were pre-treated with MBCD, there was a significant decrease in the anti-proliferative effect of bufalin (Fig. 4E). These findings collectively demonstrate that bufalin-induced apoptosis is mediated by ATP1A1 in caveolae.

### Bufalin affects the phosphorylation of Src and modulates downstream signalling pathways

**Fig. 5:**
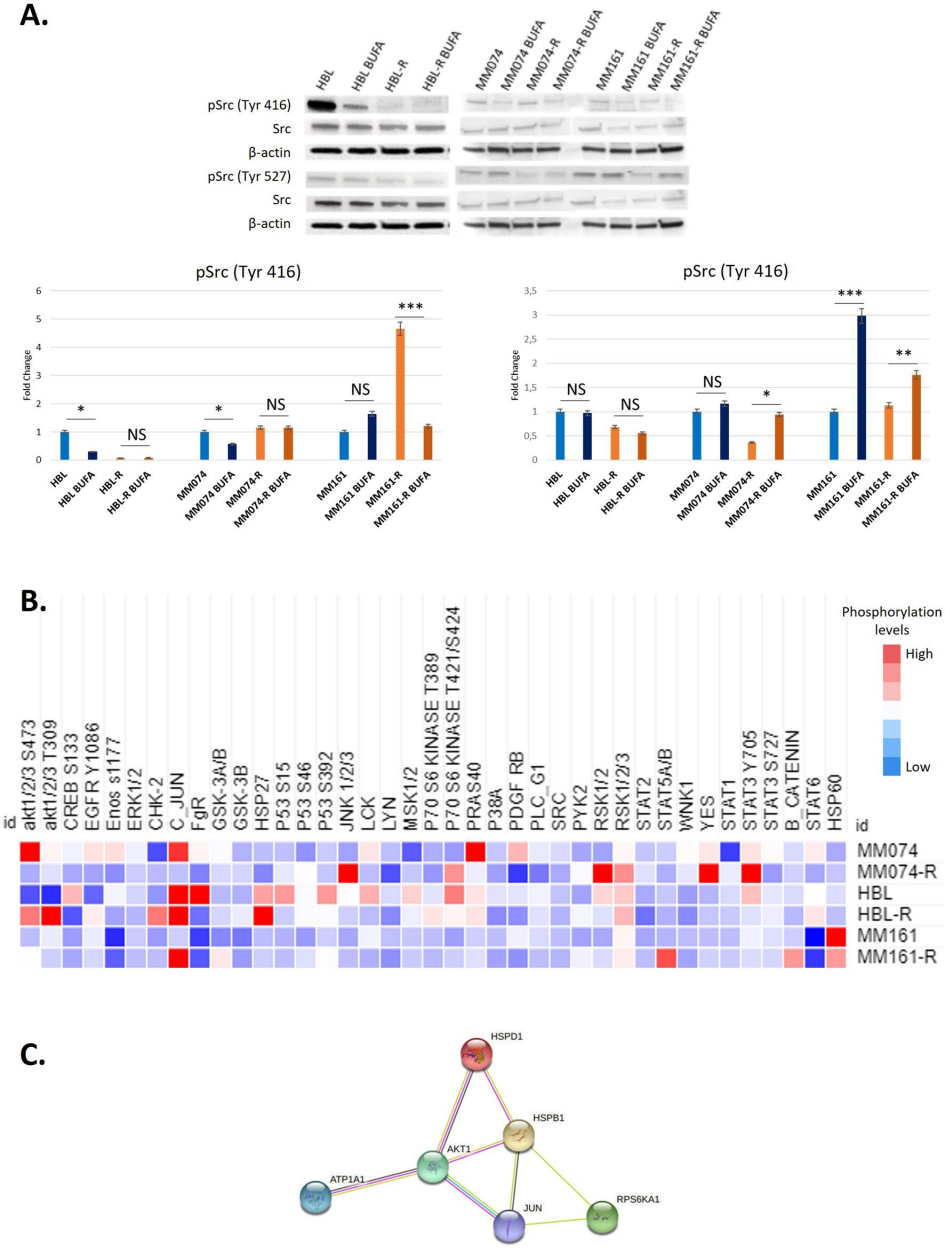
Effect of bufalin on SRC activity and signaling pathways. **A** Western blot analysis and quantification of the active form of SRC (phosphorylation 416) and the inactive form (phosphorylation 527) in melanoma cells (sensitive and resistant (-R)) treated with 10 nM bufalin (BUFA) for 24 h, data are presented in mean + SD, ANOVA and Tukey post-hoc test. **B** Phosphorylation profiles in melanoma cell lines; heatmap showing the phosphorylation level of 37 proteins according to the cell lines and the treatment (10 nM bufalin for 30 min vs untreated). Shades of red represents a high phosphorylation profile while shades of blue correspond to a weak phosphorylation expression. **C** Signaling network including the most significant correlation with ATP1A1 in relation with bufalin treatment (STRING)

Considering the localization of ATP1A1 in caveolae, we may expect an impact of the pump on the regulation of intracellular signalling pathways. Therefore, we investigated the effect of bufalin on signal transduction and specifically evaluated the phosphorylation profile of the Src protein, which is known to be downstream of ATP1A1. To assess these changes, we conducted Western blot analysis to examine the phosphorylation status of Src at Tyr416 (activation) and Tyr527 (inhibition) in cell lines treated with 10 nM bufalin for 24 h. The results revealed that bufalin induced a decrease in Tyr416 phosphorylation in HBL, MM074, and MM161-R cells, indicating a reduction in Src activation. Conversely, bufalin treatment led to an increase in Tyr527 phosphorylation in MM074-R, MM161, and MM161-R cells, suggesting enhanced inhibition of Src activity (Fig. 5A). These findings indicate that, with the exception of the HBL-R cell line, bufalin mediates its inhibitory signaling by reducing the activation phosphorylation and/or increasing the inhibition phosphorylation of the Src protein, resulting in Src inactivation.

To elucidate the signaling pathways affected by bufalin, we conducted a phosphokinase array to obtain a comprehensive overview of the phosphorylation changes induced by bufalin treatment (10 nM for 30 min) in both sensitive and resistant cell lines **(**Fig. 5B). The array encompassed the evaluation of protein phosphorylation and expression for a total of 39 proteins. We observed that bufalin treatment resulted in an overall increase in protein levels, with fold changes of 1.63 in HBL, 1.59 in HBL-R, 1.53 in MM074, 1.51 in MM074-R, 1.21 in MM161, and 1.29 in MM161-R. Notably, these increases showed a significant negative correlation with the IC50 values of the cell lines for bufalin (r = − 0.943, p = 0.005) (Table 2). This suggests that the most sensitive cell lines, namely HBL and HBL-R, exhibit the highest extent of phosphorylation changes in response to bufalin treatment. Importantly, the mRNA expression of ATP1A1 positively correlated with the overall levels of protein phosphorylation (r = 0.886, p = 0.019), and negatively correlated with the IC50 values for bufalin (r = − 0.829, p = 0.042).

Upon investigating the main changes induced by bufalin, we observed that in at least two cell lines, several proteins showed upregulation (defined as fold-change > 2) including akt1-2-3_S473, C-JUN, HSP27, P70-S6-KINASE_T421_S424, RSK1-2–3, and HSP60. These findings suggest that ATP1A1 is associated with a network involving AKT, JUN, RPS6K, and HSP (Fig. 5C). Additionally, at least two cell lines exhibited downregulation (fold-change > 2) of FgR and STAT6. Notably, paired cell lines displayed discrepancies in the variations of certain proteins. For example, akt1-2-3_S473 and akt1-2-3_T309 were downregulated in HBL but upregulated in HBL-R, CREB_S133 showed an increase in HBL but a decrease in HBL-R, and PDGF_RB exhibited enhanced expression in MM074 while reduced expression in MM074-R. Moreover, ATP1A1 mRNA levels positively correlated with changes in GSK_3B (r = 0.829, p = 0.04), P53_S15 (r = 0.829, p = 0.04), and STAT6 (r = 0.829, p = 0.04), while the IC50 value for bufalin negatively correlated with variations in STAT6 (r = − 0.943, p = 0.005).

Altogether, although bufalin induced similar cellular effects such as cell apoptosis and inactivation of Src in all tested cell lines through ATP1A1 inhibition, it exerted its effects by influencing different signaling pathways. Furthermore, when comparing paired lines (sensitive vs resistant to targeted therapy), bufalin regulated different phosphoproteins. Additionally, no significant difference (according to the Mann–Whitney test) was observed when comparing sensitive and resistant cells. These findings further suggest that bufalin acts through cell-specific mechanisms, as these lines exhibit distinct phenotypes, alternative pathways, and metabolic switches [31].

### *Bufalin reduces tumour development *in vivo

Finally, we conducted an in vivo study to assess the safety of bufalin and its impact on tumor development. In order to ensure a continuous distribution of bufalin and avoid potential issues related to its cardiotonic effects resulting from the serum peak after intraperitoneal (IP) administration, we used Alzet osmotic pumps to deliver a stable dose of 2 mg/kg/day of bufalin to the nude mice (6 animals per condition) for a duration of 14 days. Specifically, focusing on the BRAF mutant model, we xenografted MM074 or MM074-R cell lines into mice to establish tumors that are either sensitive or resistant to targeted therapy (vemurafenib). Subsequently, the animals were exposed to bufalin for a period of 14 days (Additional file 3: Fig. S3, experimental design).

**Fig. 6:**
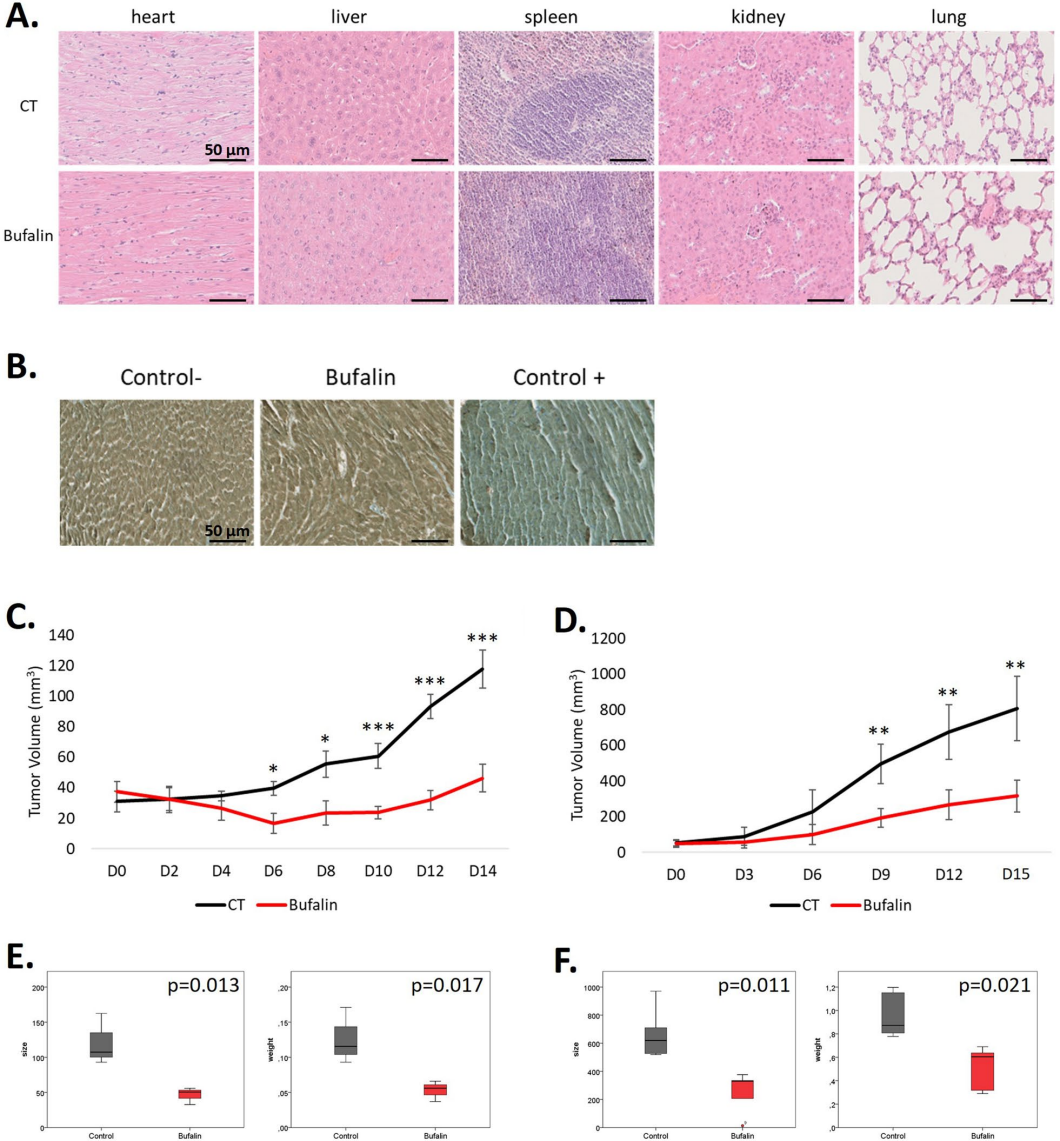
Effect of bufalin in nude mice xenografted with MM074 or MM074-R cell lines, 6 mice were for each experimental condition. **A** No effect of stably deliver 2 mg/kg/d of bufalin to the mice for 14 days regarding to heart, liver, spleen, kidney and lung tissue morphology. **B** No side effect of bufalin evaluating troponin I staining in hearts of control mice and bufalin treated mice (Control + is a heart of a mouse treated with silica to induce an autoimmune myocarditis). Scale bars in B, C = 50 µm. **C** Effect of bufalin on tumor volume developed from MM074 cells in nude mice. **D** Effect of bufalin on tumor volume growing from MM074-R cells in nude mice. Data C, D are presented in mean + SD, ANOVA and Tukey post-hoc test. **E** Box plot for MM074 cells presenting tumor volume and weight after mouse euthanasia in both control and bufalin-treated groups. **F** Box plot for MM074-R cells showing tumor volume (mm^3^) and weight (g) after mouse euthanasia in both control and bufalin-treated groups (Mann Whitney test)

Regarding the toxicity of bufalin, we monitored the body weight of the mice throughout the treatment period and compared it between the control and treated groups. Importantly, we did not observe any significant changes in body weight between these two groups (Additional file 4: Fig. S4). Furthermore, at the time of sacrifice, we collected and weighed the organs from the mice in both the control and treated groups. Subsequently, we performed Haematoxylin–Eosin staining to examine the histological structures of these organs. Encouragingly, we did not observe any noticeable impact of bufalin on the organ/body weight ratio (Additional file 4: Fig. S4) nor on the morphology of the normal tissues (Fig. 6A and Additional file 5: Fig. S5). Moreover, as bufalin is known to be a cardiotonic steroid that could potentially induce cardiac dysfunction as a side effect, we conducted troponin I staining in the hearts of control mice, bufalin-treated mice, and mice with confirmed cardiac dysfunction (used as positive controls). Importantly, we did not observe any troponin I staining in the hearts of both control mice and bufalin-treated mice, unlike the mice with documented heart dysfunction (Fig. 6B).

About tumor progression in mice, we found a significant decrease in tumor volumes in the bufalin-treated group compared to the control group. Specifically, starting from day 6, we observed a significant reduction in tumor volumes in the bufalin-treated group for the sensitive tumors (Fig. 6C). Similarly, for the resistant tumors, we observed a significant decrease in tumor volumes from day 9 in the bufalin-treated group compared to the control group (Fig. 6D). Additionally, when evaluating tumor size and weight at the time of sacrifice (Day 14), we observed a significant decrease of the tumor size and weight in the bufalin-treated mice for both sensitive tumors (Fig. 6E) and resistant tumors (Fig. 6F) compared to not treated mice.

## Discussion

The primary objective of this study was to determine the role of ATP1A1 in the prognosis of metastatic melanoma and its association with the resistance to targeted therapy. Hence, we observed a significant correlation between higher ATP1A1 expression and shorter overall survival (OS) in patients. This finding is in line with a previous study that suggested a crucial role for ATP1A1 in melanoma progression, demonstrating that elevated levels of ATP1A1 in tumors were associated with high Breslow indexes and brain metastasis development [15]. However, our study is the first to report a direct link between higher ATP1A1 levels and shorter patient survival. These findings further support the growing evidence of the involvement of Na + /K + -ATPase in cancer initiation, growth, development, and metastasis [32].

In addition, our data indicate that high ATP1A1 expression is associated with non-response to the BRAF inhibitor vemurafenib in patients and different MAPK inhibitors in cell lines. To our knowledge, this is the first study to demonstrate a link between ATP1A1 and resistance to targeted therapy. However, previous studies in other contexts have reported associations between ATP1A1 and chemoresistance [33, 34], as well as ATP1A1 and senescence, which could potentially play a role in therapy resistance [35, 36]. Since our data were obtained from a small cohort, it will be necessary to confirm these findings with a larger population to establish whether ATP1A1 levels could be routinely evaluated to better define which melanoma patients would benefit from targeted therapy.

Investigating 448 cutaneous melanomas from the TCGA PanCancer Atlas, we discovered a positive correlation between high ATP1A1 mRNA expression and markers of differentiation and pigmentation (MITF, SOX10, TYR, MLANA). Additionally, through transcriptome profiling of 11 melanoma cell lines, we found the presence of ATP1A1 in the Verfaillie proliferative gene signature, which is associated with the melanocytic state characterized by MITF and SOX10 expression. These findings further support ATP1A1 as a target gene of MITF [37] and underscore its relevance as a potential target for melanoma treatment.

Then, we assessed the impact of bufalin, one of the natural ligands of ATP1A1, on sensitive and resistant melanoma cell lines both in vitro and in vivo. Bufalin is an endogenously expressed [21] cardiotonic steroid known to interact with ATP1A1 [38]. Our in vitro findings demonstrated that bufalin at nanomolar concentrations, which are approximately 100 times lower than the theoretical safety doses for cardiac injury [16], reduced clonogenicity and induced apoptosis in melanoma cells. It is worth noting that bufalin can become toxic when it affects the pump function of the Na + /K + ATPase. However, research has shown that at low concentrations, the cardiotonic steroid blocks the signal transduction function of ATP1A1 located in caveolae without affecting its pump activity [39]. Although a few studies have already explored the role of bufalin in melanoma and demonstrated its ability to induce apoptosis [40], our study is the first to reveal that bufalin exhibits stronger anti-cancer effects in melanoma cells resistant to targeted therapy. This could be attributed to the upregulation of ATP1A1 expression in resistant cells, making them more susceptible to bufalin. Notably, the resistant cells exhibited a more differentiated phenotype, which is associated with the activation of MITF, a transcription factor for ATP1A1 [37]. Previous studies have highlighted the potential of bufalin in increasing the sensitivity of cancer cells to targeted therapy, particularly sorafenib in hepatocellular carcinoma [41–43] and MEKi in leukemia [44], as well as in reversing ABCB1-mediated resistance to docetaxel in breast cancer [58]. Therefore, bufalin may potentially delay the development of resistance or restore sensitivity in metastatic melanoma patients undergoing targeted therapy, as evidenced in our study, or chemotherapy.

From a mechanistic point of view, our knock-down experiments and disruption of caveolae validated ATP1A1 in caveolae as the main target of bufalin in melanoma. Our previous study has already established a connection between ATP1A1 levels and sensitivity to bufalin [22], and similar findings have been observed in glioma [45, 46]. Furthermore, our proximity ligation assay (PLA) analyses provided evidence of a physical interaction between ATP1A1 and Cav-1, confirming the presence of a consensus Cav-1 binding motif on ATP1A1 [47]. In addition, our Western blotting and phosphoprotein assays revealed the inhibitory effect of bufalin on Src activity, leading to the disruption of signal transduction. Indeed, bufalin has been reported to regulate pleiotropically a myriad of signal transduction cascades in various cancers [59]. The involvement of the ATP1A1-Cav1-Src complex in the inhibitory activity of cardiotonic steroids on signaling pathways has been demonstrated in various cancer types [9, 16, 48–51], and now we have established its significance in melanoma.

Of note, in addition to its action on the ATP1A1 isoform, bufalin also modulates the translation of cancer-related proteins by indirectly acting on eIF4A and eIF4G through the strong inhibition of the transcriptional co-activator SRC-3 and of the transcription factor cMYC in triple-negative breast cancer [60]. Of note, the eIF4A factor is involved in acquired resistance of melanoma cells to MAPK inhibitors (MAPKi) [61, 62]. Following bufalin treatment, cancer cells reorganize the translation of specific mRNAs involved, notably, in the mTOR pathway that contributes to the emergence of a population of cells insensitive to MAPKi, also known as persistent cells. Recently, new potential direct interaction proteins of bufalin were screened using a human proteome microarray containing 21,838 human proteins, revealing the Bifunctional Apoptosis Regulator (BFAR) as a new direct target protein of bufalin, with several potential binding sites for the steroid. BFAR has anti-apoptotic activity, both for apoptosis triggered via death-receptors and via mitochondrial factors. It is highly expressed in gastric cancer (GC) and promotes the occurrence and metastasis of GC by activating the PI3K/AKT/mTOR signaling pathway both in vitro and in vivo. Bufalin reversed the promoting effect of BFAR on the carcinogenesis and metastasis of GC by downregulating the expression of BFAR [63]. Hence, prospective studies should aim to examine SRC-3 and BFAR as potential new targets for melanoma treatment through their inhibition by bufalin.

## Conclusions

To conclude, our study highlights the potential of ATP1A1 as a valuable prognostic marker for metastatic melanoma patients, as well as a predictive marker for the response to targeted therapy. Based on our findings, ATP1A1 emerges as a promising target for melanoma treatment, and its inhibition by bufalin could be considered to reduce cancer progression by overcoming resistance to MAPK inhibitors. However, considering the narrow therapeutic window of cardiotonic steroids, further research is needed to determine bufalin doses that effectively inhibit the signaling pathway activity of ATP1A1 without affecting its pump function. In this context, since bufalin is an endogenous steroid detected at lower concentrations in the serum of cancer patients compared to healthy individuals [21], bufalin supplementation could potentially be beneficial for cancer patients in restoring normal serum levels and improving treatment outcomes.

### Supplementary Information

**Additional file 1: Figure S1.** Evaluation of ATP1A1 mRNA in 17 types of cancers including melanoma (TCGA dataset using The Human Protein Atlas).

**Additional file 2: Figure S2.** Comparison of the expression levels of the 4 isoforms of ATP1A showing that only ATP1A1 is significantly different between responders and non-responders.

**Additional file 3: Figure S3.** MM074-R tumor control (**A**), MM074-R bufalin-treated tumor (**B**), Alzet osmotic pump (**C**) in mice.

**Additional file 4: Figure S4.** No change in the mouse body weight between control and treated groups throughout the treatment. No impact of bufalin on the organ/body weight ratio (p>0.05).

**Additional file 5: Figure S5.** Uncropped western blots. **A** Actin HBL(-R). **B** Total Src MM074 and MM161 **C** p-Src (Tyr527) MM074 and MM161 **D**.

## Data Availability

All the data are available in the supplementary materials. The data underlying this article will be shared on reasonable request to the corresponding authors.
