# Supplementary figures and images for: ATP1A1 is a promising new target for melanoma treatment and can be inhibited by its physiological ligand bufalin to restore targeted therapy efficacy
Source: Cancer Cell Int. 2024 Jan 4;24:8. doi: 10.1186/s12935-023-03196-y (PMC10765859; doi:10.1186/s12935-023-03196-y)

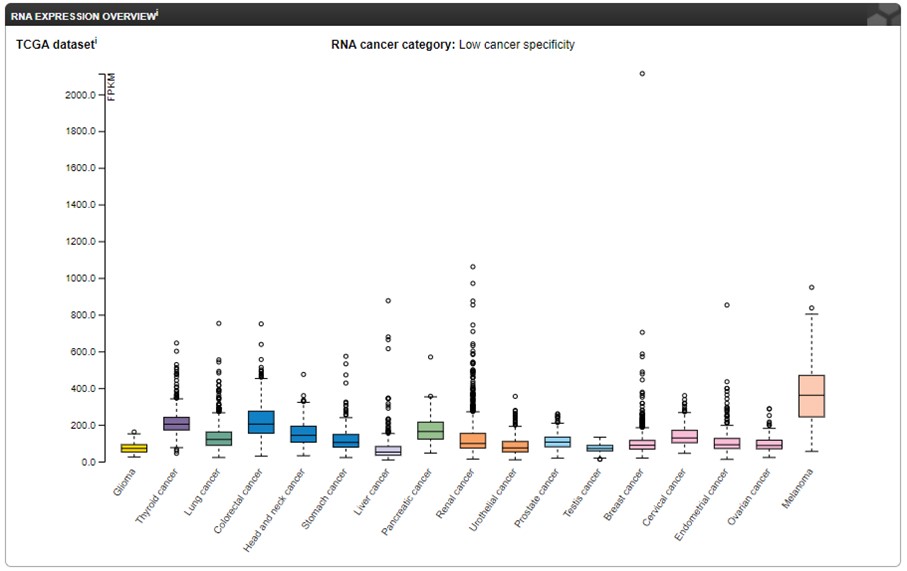

Supplement: Supplementary file 1 — Additional file 1: Figure S1. Evaluation of ATP1A1 mRNA in 17 types of cancers including melanoma (TCGA dataset using The Human Protein Atlas). [file 12935_2023_3196_MOESM1_ESM.jpg]

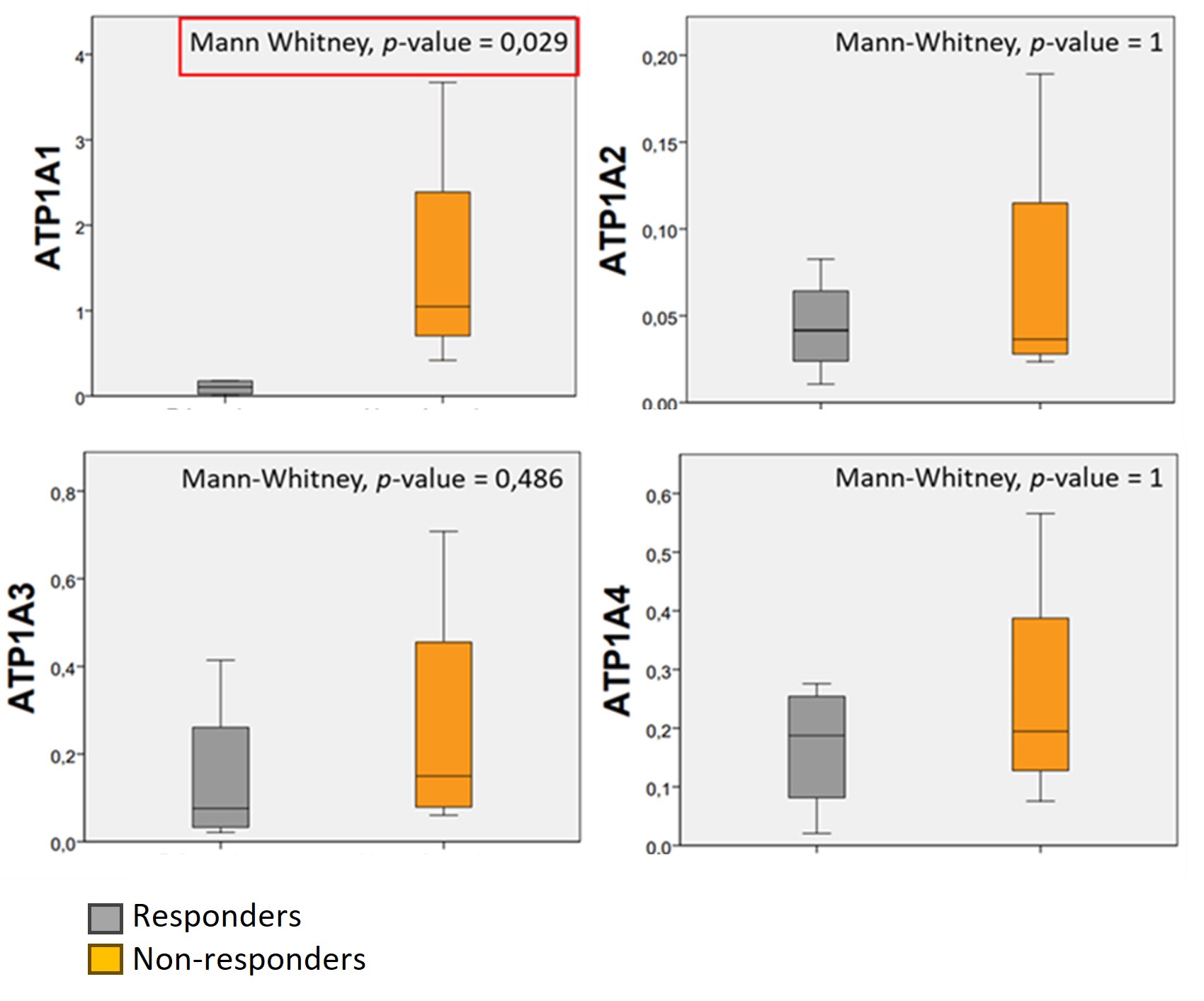

Supplement: Supplementary file 2 — Additional file 2: Figure S2. Comparison of the expression levels of the 4 isoforms of ATP1A showing that only ATP1A1 is significantly different between responders and non-responders. [file 12935_2023_3196_MOESM2_ESM.jpg]

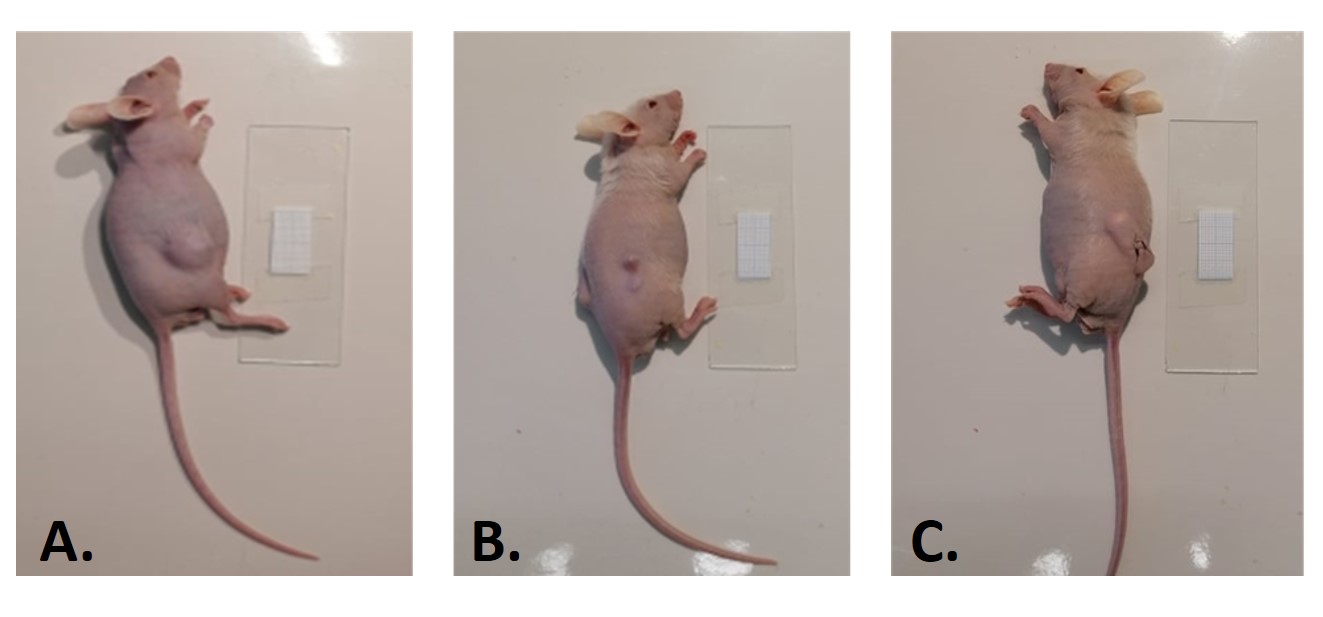

Supplement: Supplementary file 3 — Additional file 3: Figure S3. MM074-R tumor control (A), MM074-R bufalin-treated tumor (B), Alzet osmotic pump (C) in mice. [file 12935_2023_3196_MOESM3_ESM.jpg]

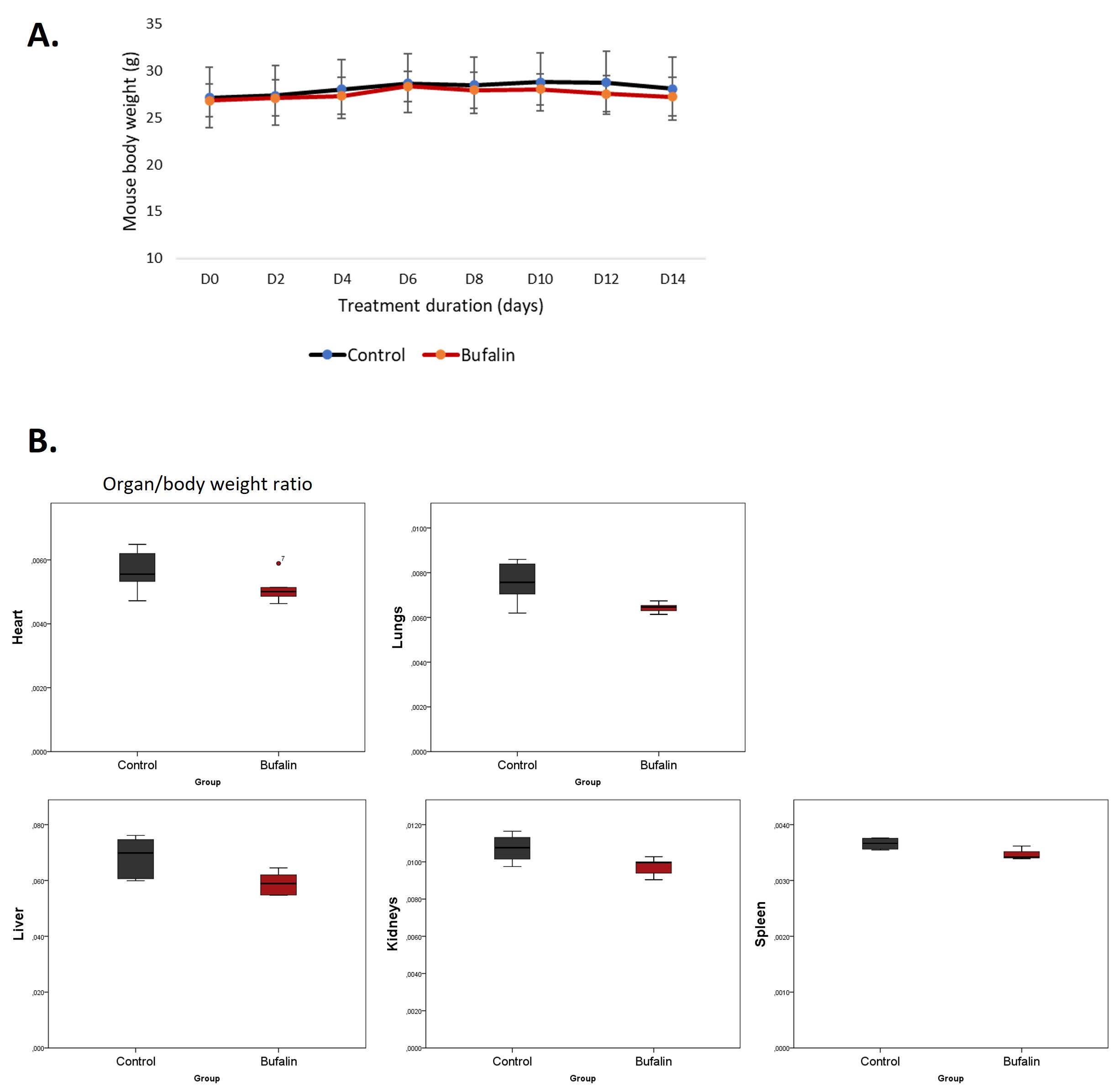

Supplement: Supplementary file 4 — Additional file 4: Figure S4. No change in the mouse body weight between control and treated groups throughout the treatment. No impact of bufalin on the organ/body weight ratio (p>0.05). [file 12935_2023_3196_MOESM4_ESM.jpg]

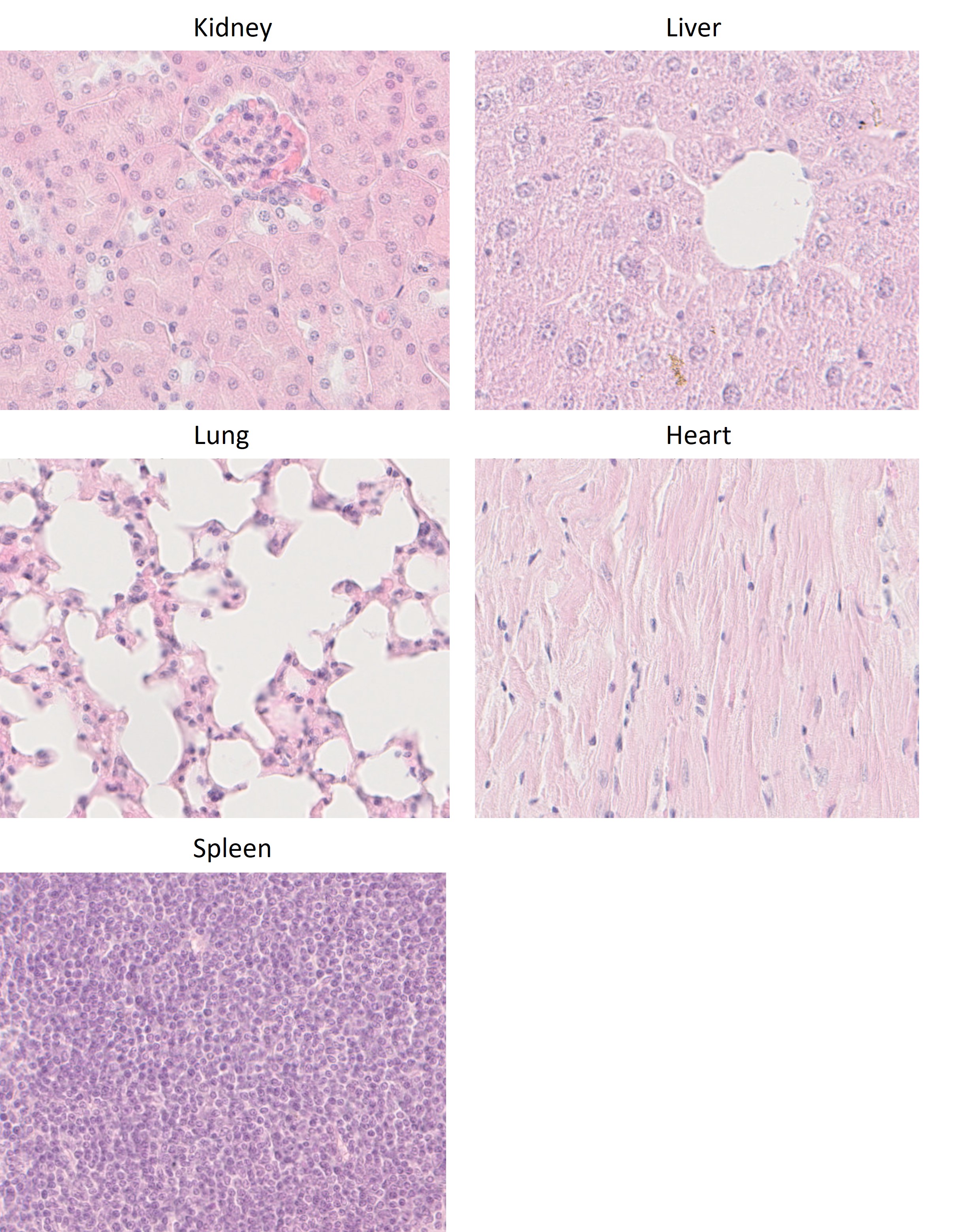

Supplement: Supplementary file 5 — Additional file 5: Figure S5. Uncropped western blots. A Actin HBL(-R). B Total Src MM074 and MM161 C p-Src (Tyr527) MM074 and MM161 D. [file 12935_2023_3196_MOESM5_ESM.jpg]
